# Sorafenib and triptolide loaded cancer cell-platelet hybrid membrane-camouflaged liquid crystalline lipid nanoparticles for the treatment of hepatocellular carcinoma

**DOI:** 10.1186/s12951-021-01095-w

**Published:** 2021-11-08

**Authors:** Zhe Li, Gang Yang, Lu Han, Rong Wang, Chunai Gong, Yongfang Yuan

**Affiliations:** Department of Pharmacy, Shanghai 9th People’s Hospital, Shanghai Jiao Tong University School of Medicine, No. 280 Mohe Road, Shanghai, 201999 China

**Keywords:** Sorafenib, Triptolide, Biomimetic nanoparticles, Hepatocellular carcinoma

## Abstract

In addition to early detection, early diagnosis, and early surgery, it is of great significance to use new strategies for the treatment of hepatocellular carcinoma (HCC). Studies showed that the combination of sorafenib (SFN) and triptolide (TPL) could reduce the clinical dose of SFN and maintain good anti-HCC effect. But the solubility of SFN and TPL in water is low and both drugs have certain toxicity. Therefore, we constructed a biomimetic nanosystem based on cancer cell-platelet (PLT) hybrid membrane camouflage to co-deliver SFN and TPL taking advantage of PLT membrane with long circulation functions and tumor cell membrane with homologous targeting. The biomimetic nanosystem, SFN and TPL loaded cancer cell-PLT hybrid membrane-camouflaged liquid crystalline lipid nanoparticles ((SFN + TPL)@CPLCNPs), could simultaneously load SFN and TPL at the molar ratio of SFN to TPL close to 10:1. (SFN + TPL)@CPLCNPs achieved long circulation function and tumor targeting at the same time, promoting tumor cell apoptosis, inhibiting tumor growth, and achieving a better "synergy and attenuation effect", which provided new ideas for the treatment of HCC.

**Supplementary Information:**

The online version contains supplementary material available at 10.1186/s12951-021-01095-w.

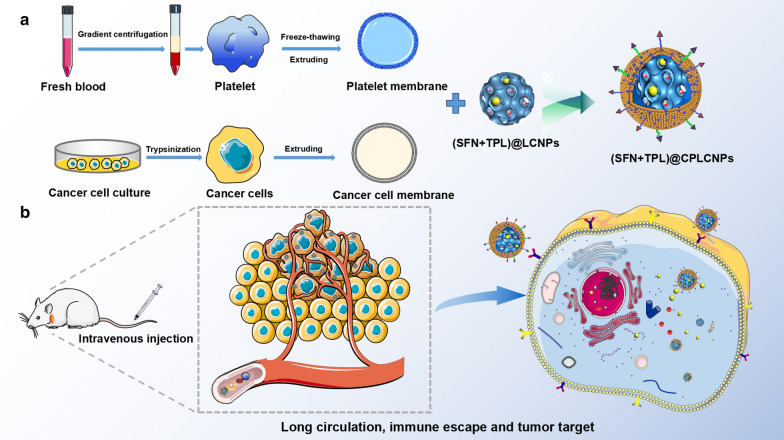

## Introduction

Hepatocellular carcinoma (HCC) accounts for 90% of primary liver cancers. Worldwide, liver cancers rank fourth in the cause of tumor-related deaths, and the five-year survival rate is only 18% [1–3]. HCC can be treated by surgical resection, liver transplantation, liver-oriented therapy, and systemic chemotherapy. Among these treatment strategies, only surgical resection and liver transplantation are considered as potentially possible cures. However, more than 80% of patients unfortunately encounter advanced HCC when diagnosed, and lose the opportunities for surgical resection and liver transplantation. Therefore, in addition to early detection, early diagnosis and early surgery, it is necessary to find new strategies to improve the therapeutic effect of HCC. Combination therapy, a combined use of multiple therapeutic agents or different therapeutic methods, has been adopted to overcome the limitation of the conventional approaches for the treatment of cancer. Combination chemotherapy is the most common therapeutic combination strategy in clinic, which has shown great successes with enhanced therapeutic effects [4]. Based on our previous study and predecessors' research, we found that the combination of sorafenib (SFN) and triptolide (TPL) had shown synergistic effects on HCC [3]. However, the drug dose ratio at tumor site is uncontrollable and can't be fixed at the optimal synergistic ratio owning to a corresponding differential pharmacokinetic profile and biodistribution [5]. Moreover, the solubility of SFN and TPL in water is low and both drugs have certain toxicity [6–8]. The nano drug co-delivery system (NDCDS) is expected to solve the problems above with minimized side effects and optimized therapeutic efficacy [9].

In recent years, lyotropic liquid crystalline lipid nanoparticles (LCNPs) have emerged as a new material for drug delivery, with the merits of enhanced colloidal stability, sustained release profile, flexible structure, self-assembling properties and ability to efficiently encapsulate hydrophilic, hydrophobic, or amphiphilic drugs [10, 11]. Currently, biomimetic cell membrane-camouflaged drug delivery systems with enhanced biocompatibility, low immunogenicity and active targeting abilities have also attracted much attention to facilitate nanomedicines for biomedical applications [12]. Lots of different cell membrane-camouflaged nanoparticles, including erythrocyte, cancer cell, leukocyte, stem cell, platelet (PLT) and so on, have been widely researched. In this work, we fused PLT membrane with Huh-7 cell (human liver cancer cell line) membrane and fabricated SFN and TPL loaded cancer cell-PLT hybrid membrane-camouflaged liquid crystalline lipid nanoparticles ((SFN + TPL)@CPLCNPs) for the treatment of HCC. The cancer cell-PLT hybrid membrane vesicles were supposed to retain their parent membrane proteins and could synchronously endow the nanoparticles with long circulation derived from PLT membrane and homologous tumor targeting derived from Huh-7 cell membrane.

In this study, we designed (SFN + TPL)@CPLCNPs to co-encapsulate SFN and TPL for a synergistic anti-tumor effect. (SFN + TPL)@CPLCNPs were expected to prolong the circulation of SFN and TPL, and increase the concentration of the two drugs in tumor site. The anti-tumor activity of (SFN + TPL)@CPLCNPs and its mechanisms were also investigated in vitro and in vivo.

## Materials and methods

### Materials, cell culture, and animals

TPL and SFN (purity ≥ 98%) were obtained from Shanghai Yuanye Biotech Co., Ltd. (Shanghai, China). Coumarin-6 (C6) was supplied by Sigma-Aldrich Co. (St.Louis, MO, USA). Glyceryl monooleate (MO) was kindly donated by Gattefossé Co. (Lyon, France). Acetonitrile, ethanol and other reagents with analytical grade were purchased from Sinopharm Chemical Reagent Co., Ltd. (Shanghai, China). Cell counting kit-8 (CCK-8) was obtained from Beyotime Biotechnology (Shanghai, China). Fetal bovine serum (FBS) and Dulbecco’s modified Eagle’s medium (DMEM) were obtained from Gibco Inc. (Grand Island, NY, USA). TUNEL apoptosis assay kit was obtained from Roche Pharmaceutical Co., Ltd. (Basel, Switzerland). All other chemicals used were of analytical grade.

The Huh-7 cell line and RAW 264.7 cell line were purchased from the Cell Bank of Typical Culture Preservation Committee of the Chinese Academy of Sciences (Shanghai, China). Huh-7 cells and RAW 264.7 cells were cultured in DMEM supplemented with 10% FBS, 1% penicillin/streptomycin in a humidified incubator with 5% CO_2_ at 37 °C.

Healthy male Balb/c-nu mice (18 ± 2 g) were randomly assigned to different groups. The experiment was approved by the Ethics Committee of Ninth People’s Hospital, affiliated with Shanghai Jiao Tong University School of Medicine before the research.

### HPLC assay

The HPLC experiment was carried out on a Waters e2695 HPLC system (Waters Technologies, USA) with an Agilent TC-C18 column (250 mm × 4.6 mm, 5 μm) for the simultaneous detection of SFN and TPL. The mobile phase was a mixture of acetonitrile and water (70:30, v/v), and the flow rate was set at 1.0 mL·min^−1^ with an injection volume of 20 μL. The detection wavelength was 225 nm with the column temperature maintained at 25 °C. All the reagents used were HPLC grade. The HPLC method was validated for the detection of SFN and TPL.

### *Preparation of (SFN* + *TPL)@CPLCNPs*

(SFN + TPL)@LCNPs were prepared with the emulsification method. In this method, MO (4%, w/v), drugs and P407 (0.5%, w/v) were melted in a water bath at 70 °C. The molten mixture was then added dropwise into water preheated to 70 °C under magnetic stirring for 15 min. The mixtures were then sonicated on a probe sonicator at 30% amplitude with a 5-s on, 5 s-off circle for 3 min to form a uniform opaque mixture [11]. Cancer cell and PLT membranes were separated using procedures reported previously [13–16]. To prepare PLT membrane-camouflaged LCNPs (PLCNPs), PLT membrane and LCNPs were mixed at the mass ratio of 1.0 in PBS and subsequently sonicated for 2 min at a power of 100 W [17]. Similarly, we prepared cancer cell membrane-camouflaged LCNPs (CLCNPs). To prepare cancer cell-PLT hybrid membrane-camouflaged LCNPs (CPLCNPs), cancer cell membrane was mixed with PLT membrane at mass ratio of 2:1 and added to LCNPs. Then, the mixtures were sonicated for 2 min at a power of 100 W. All the NP samples were stored at 4 °C for further use. To determine the drug loading (DL) capacity, the ultrafiltration method was adopted [18]: 100 μL preparation was added with methanol (preparation:methanol = 1:9, V/V) and sonicated for 10 min to destroy the nanostructure. The solution was then filtered through a 0.45 μm membrane, and the contents of SFN and TPL in the preparation were determined by HPLC. Another 100 μL preparation was precisely removed into a 100 kDa ultrafiltration tube, followed by centrifugation at 10,000 rpm. The filtrate was also detected on HPLC to determine the free SFN and TPL. The DL was calculated with formula 1:

1 $$DL\% = \frac{{W_{T} - W_{F} }}{{W_{E} }} \times {1}00\%$$

where *W*_*T*_ was the total drug in the preparations, *W*_*F*_ was the free drug in the filtrate and *W*_*E*_ was the total weight of excipients used in the preparations.

### Characterization of the prepared nanoparticles

The particle size, polydispersity index (PDI) and zeta potential of the nanoparticles were measured on a Malvern ZS90 Zetasizer (Malvern, Worcesterchire, UK). The stability of the nanoparticles were also evaluated. The morphology of the membrane-camouflaged nanoparticles was observed by a JEM-1011 transmission electron microscope (TEM) instrument (JEOL, Ltd., Tokyo, Japan). Proteins on the nanoparticles were characterized using SDS-PAGE.

### In vitro* release*

The drug release profiles of (SFN + TPL)@CPLCNPs, (SFN + TPL)@LCNPs and free (SFN + TPL) were evaluated with a dialysis method. 2 mL of (SFN + TPL)@CPLCNPs, (SFN + TPL)@LCNPs or free (SFN + TPL) suspension (containing 1 mg SFN) was loaded into a dialysis bag and immersed in 100 mL of release buffer (PBS containing 0.1% w/v Tween 80, pH 5.5) with shaking (100 rpm) at 37 ℃. At predetermined time points, 1 mL of the external medium was withdrawn and replaced with an equal volume of fresh pre-heated medium. The concentration of SFN or TPL in the release medium was analyzed on HPLC [19].

### In vitro* cellular uptake studies*

The in vitro cellular uptake of the nanoparticles was respectively evaluated with Huh-7 cancer cells and RAW 264.7 macrophage cells. Cancer cell-PLT hybrid membrane-camouflaged liquid crystalline lipid nanoparticles (CPLCNPs) were labeled with C6. The cellular uptake of the nanoparticles was evaluated by a Nikon A1 (Nikon, Japan) confocal laser scanning microscopy (CLSM) and a FACScanto flow cytometry (BD, USA). For CLSM observation, Huh-7 cells or RAW 264.7 macrophage cells were seeded into a glass-bottom Petri dish at the density of 5 × 10^4^ cells per well 24 h prior to the experiment. The cells were then incubated with C6 labeled LCNPs, CLCNPs, PLCNPs or CPLCNPs at 37 ℃ for 1.5 h. After that, the C6 labeled nanoparticles were removed. Afterwards, the cells were rinsed three times with PBS and fixed with 4% paraformaldehyde, followed by washing three times with PBS and staining with Hoechst 33,258 for 5 min. At last, the cells were rinsed three times with PBS and observed by CLSM. For flow cytometric analysis, Huh-7 cells or RAW 264.7 macrophage cells were seeded into 6-well plates at the density of 2 × 10^5^ cells per well for 24 h prior to the experiment. The cells were then incubated with C6 labeled LCNPs, CLCNPs, PLCNPs or CPLCNPs at 37 ℃ for 1.5 h. Then, the C6 labeled nanoparticles were removed. Afterwards, the cells were rinsed three times with PBS, followed by digesting with trypsin, collecting by centrifugation, and washing three times with PBS. Finally, the cells were resuspended in PBS for flow cytometry quantification [20].

### In vitro* cytotoxicity and apoptosis studies of (SFN* + *TPL)@CPLCNPs*

SFN/TPL with molar ratio at 10:1 was selected as best drug combination for the treatment of HCC (supplementary material Table S1). To investigate the in vitro anti-tumor activity of (SFN + TPL)@CPLCNPs, in vitro cytotoxicity and apoptosis analyses were evaluated.

#### Cytotoxicity

The cytotoxicity of the nanoparticles towards Huh-7 cells was carried out with a CCK-8 assay. Briefly, Huh-7 cells were seeded into 96-well plates at a density of 5 × 10^4^/mL and cultured overnight. Then, the culture medium was removed. Next, the cells were co-incubated with various concentrations of SFN and TPL-loaded nanoparticles for 24 h. Then, 10 μL CCK-8 was added to each well and incubated for another 2 h. The absorbance was measured at the wavelength of 450 nm by a microplate reader (Biotek, USA). The cell inhibition rate was calculated by formula 2:

2 $$Cell growth inhibition rate \left( \% \right) = \left( {1 - \frac{{OD_{E} - OD_{B} }}{{OD_{C} - OD_{B} }}} \right) \times 100\%$$

where *OD*_*E*,_ *OD*_*C*_ and *OD*_*B*_ were the absorbance of experimental group, control group and blank group, respectively. Calculations of the 50% inhibitive concentration (IC_50_) and combination index at 50% inhibitive concentration (CI_50_) were performed on a CompuSyn software (Biosoft, UK).

#### Apoptosis analyses

For apoptosis analysis, Huh-7 cells were seeded into 6-well plates at a density of 2 × 10^6^/mL and cultured overnight. Then, the culture medium was removed. Next, the cells were co-incubated with SFN and TPL-loaded nanoparticles (various formulations at an equivalent TPL concentration of 20 nM) for 24 h. At last, the cells were collected after digesting with trypsin, stained with annexin V-FITC and propidium iodide (PI) and resuspended in 500 μL of binding buffer. The samples were analyzed by flow cytometry.

### In vivo* biodistribution of homologous-targeting (SFN* + *TPL)@CPLCNPs*

Male Balb/c-nu mice were injected with 2 × 10^6^ Huh-7 cells into the left axillary region of each mouse [21]. The tumor volume and weight of the tumor-bearing mice were recorded every two days. For in vivo biodistribution study, the tumor-bearing mice were randomly divided into LCNPs, CLCNPs, PLCNPs or CPLCNPs group and intravenously injected with Cyanine 5.5 NHS ester-labeled nanoparticles via tail vein. The mice were then anesthetized and the in vivo biodistribution of the Cyanine 5.5 NHS ester-labeled nanoparticles in the mice were observed on a real-time in vivo fluorescence animal imager (Caliper IVIS Lumina II, Xenogen, USA) with the excitation wavelength at 678 nm. The fluorescence distribution of removed tissues was also evaluated.

### In vivo* anti-tumor activity of (SFN* + *TPL)@CPLCNPs*

For in vivo anti-tumor activity, the tumor-bearing nude mice were randomly divided into saline group, SFN@CPLCNPs group, TPL@CPLCNPs group, (SFN + TPL) injection group, (SFN + TPL)@LCNPs group, (SFN + TPL)@CLCNPs group, (SFN + TPL)@PLCNPs group, (SFN + TPL)@CPLCNPs group. Each animal in those groups received 5 times IV injection at an equivalent TPL dose of 0.5 mg/kg in 10 days. The bodyweights and tumor volumes of those mice were recorded every two days. The mice were then sacrificed 2 days after the last injection, and the tumors were excised, weighed and analyzed by TUNEL staining assay.

### Statistical analysis

The data were presented as mean ± standard deviation (SD). The significance of differences was evaluated with one-way ANOVA, which was carried out with SPSS 17.0 (SPSS Inc., USA). *p* < 0.05 was used as evaluation criteria of significance.

## Results and discussion

### *Characterization of (SFN* + *TPL)@CPLCNPs*

**Fig. 1:**
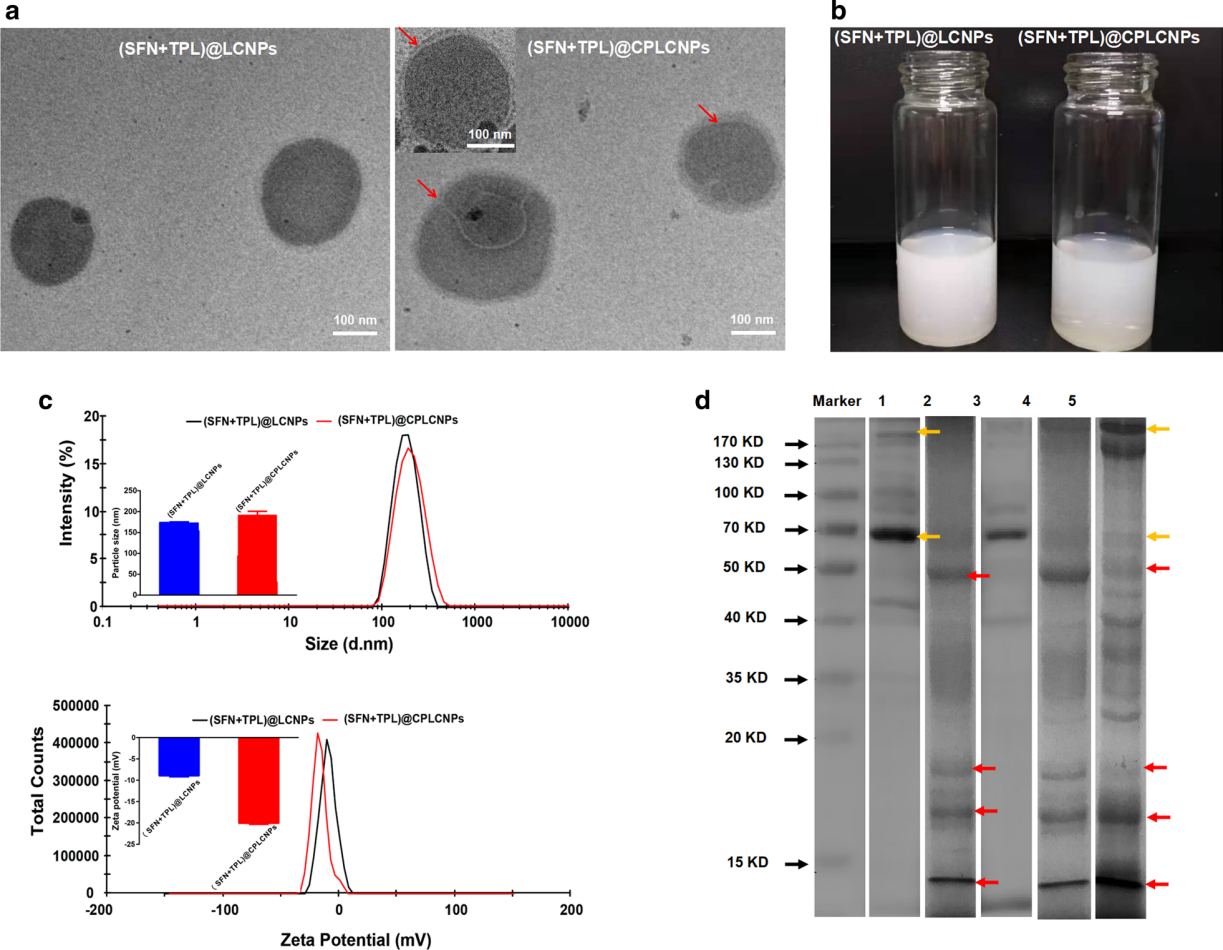
Characterization of the nanoparticles. **a** The TEM micrographs of (SFN + TPL)@LCNPs and (SFN + TPL)@CPLCNPs. **b** The appearance of (SFN + TPL)@LCNPs and (SFN + TPL)@CPLCNPs. **c** The size distribution and zeta potential of (SFN + TPL)@LCNPs and (SFN + TPL)@CPLCNPs **d** Protein profiles of 1:platelet, 2:Huh-7 cell, 3:SFN/TPL@PLCNPs, 4:SFN/TPL@CLCNPs, and 5:SFN/TPL@CPLCNPs assessed using SDS-PAGE electrophoresis

(SFN + TPL)@LCNPs were prepared with the emulsification method. To realize long circulation and homologous tumor targeting of the nanoparticles, (SFN + TPL)@LCNPs were camouflaged with cancer cell-PLT hybrid membrane. The DL of SFN and TPL in (SFN + TPL)@CPLCNPs was 1.78 and 0.14%, respectively. The molar ratio of SFN to TPL was close to 10:1. As observed by the TEM (Fig. 1a), the prepared (SFN + TPL)@LCNPs and (SFN + TPL)@CPLCNPs were spherical in shape with good monodispersity. The preparations were milk-white (Fig. 1b). After hybrid membrane camouflage, (SFN + TPL)@CPLCNPs exhibited a typical core–shell structure when compared with (SFN + TPL)@LCNPs. The particle sizes of (SFN + TPL)@LCNPs and (SFN + TPL)@CPLCNPs were 174.0 ± 1.6 and 192.9 ± 8.1 nm, and Zeta potentials of (SFN + TPL)@LCNPs and (SFN + TPL)@CPLCNPs were -8.9 ± 0.2 and -20.1 ± 0.3 mV with good stability (Fig. 1c and Additional file 1: Fig. S1). The increase in particle size and decrease in Zeta potential may be attributed to the camouflaged negative-charged hybrid membrane with thickness of about 10–20 nm [12, 15, 22]. To further verify the success of hybrid membrane camouflage, proteins on the nanoparticles were characterized with SDS-PAGE. The results of SDS-PAGE showed that the (SFN + TPL)@CPLCNPs retained the characteristic proteins inherited from PLT membrane (yellow arrows in Fig. 1d) and Huh-7 cell membrane (red arrows in Fig. 1d) [17].

### In vitro* release*

**Fig. 2:**
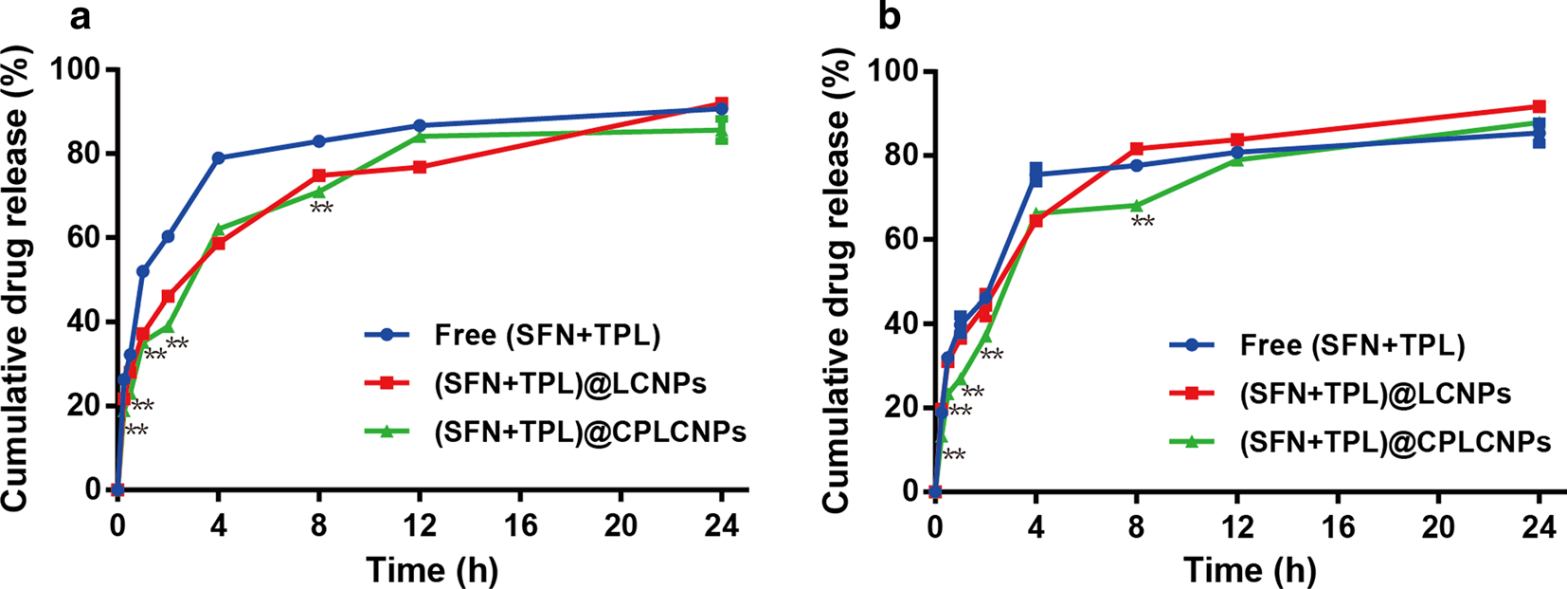
The in vitro release curves of **a** SFN and **b** TPL from free (SFN + TPL), (SFN + TPL)@LCNPs and (SFN + TPL)@CPLCNPs (n = 3; **p < 0.01 when (SFN + TPL)@CPLCNPs compared with free (SFN + TPL) or (SFN + TPL)@LCNPs)

Drug release data in PBS containing 0.1% w/v Tween 80 at pH 5.5 (simulating the slightly acidic tumor pH) were shown in Fig. 2. The release rates of SFN and TPL could be divided into two stages: the initial burst release within 2 h and the slow release from 2 to 24 h. The cumulative release percentages of SFN and TPL from free (SFN + TPL), (SFN + TPL)@LCNPs and (SFN + TPL)@CPLCNPs at 24 h were 90.7% and 85.4%, 91.9% and 91.7%, and 85.6% and 87.9%. The percentage of cumulative drug release rate at the whole time range did not show much difference. SFN and TPL could maintain the simultaneous releasing profile, which was in accordance with the optimized ratio for synergistic effect. However, the release of SFN and TPL from (SFN + TPL)@CPLCNPs exhibited slower release behaviour at 0.25, 0.5, 1, 2 and 8 h (*p* < 0.01), which might be ascribed to the hybrid membrane camouflage at the exterior of the particles blocking drug release to some extent.

### In vitro* cellular uptake studies*

**Fig. 3:**
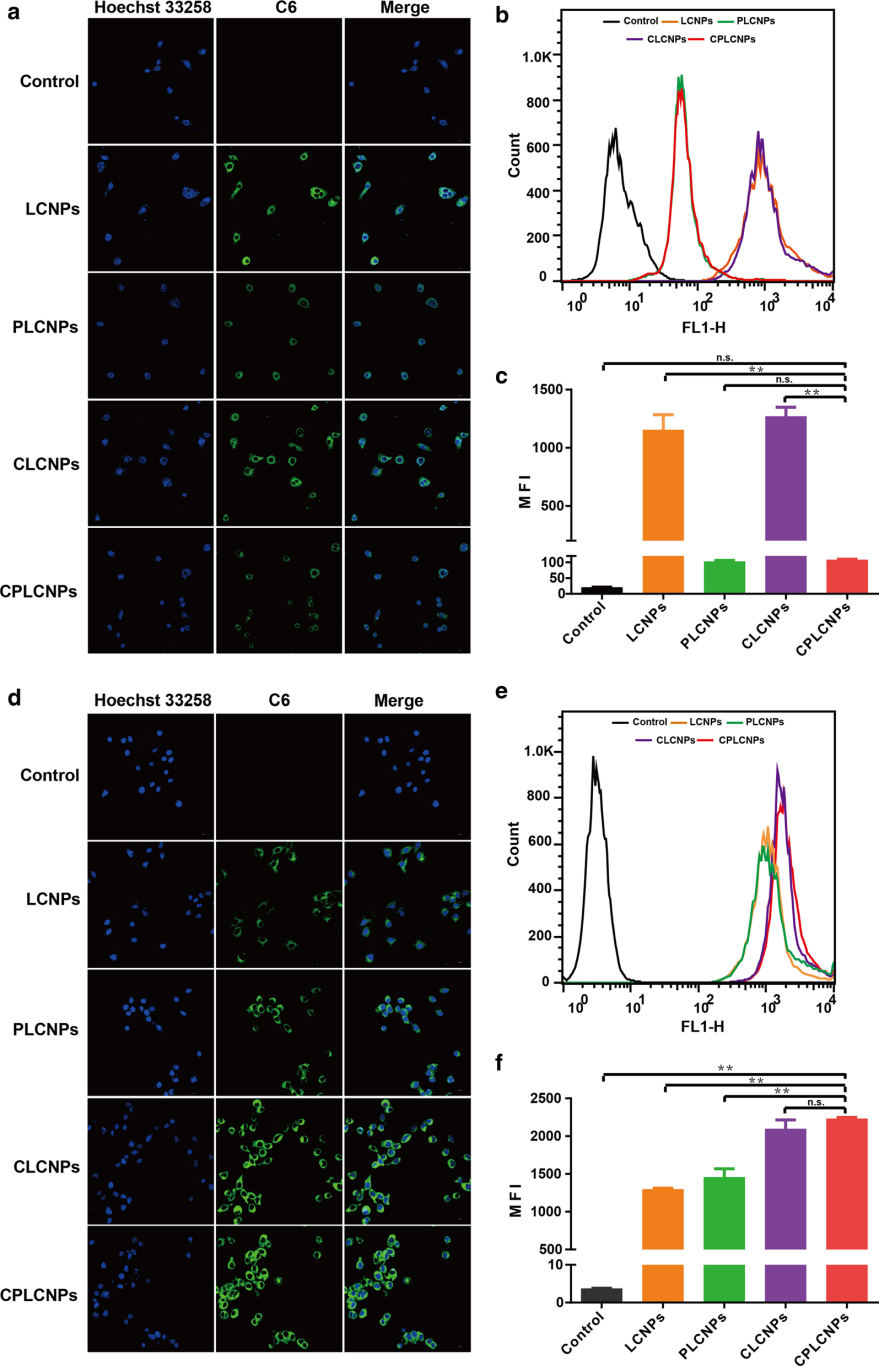
Cellular uptake of C-6-labeled formulations by RAW 264.7 cells: **a** confocal microscopy images, **b** flow cytometry, **c** the statistical results of flow cytometry and and Huh-7 cells: **d** confocal microscopy images, **e** flow cytometry, **f** the statistical results of flow cytometry (n = 3; **p < 0.01)

To evaluate the homologous tumor targeting and long circulation effects, NPs were explored by cellular uptake experiments. The delivery efficacy of NPs firstly depended on their capability to avoid mononuclear phagocyte system clearance. RAW 264.7 macrophage cell was a major component of the immune defense system [23]. The effect of cancer cell-PLT hybrid membrane camouflage on cellular uptake was assessed in murine RAW 264.7 macrophage cells by visualized LCSM and quantified FACS analysis to evaluate the immune-evasion capability. The NPs was labeled with hydrophobic fluorescence dye C6. The LCSM images showed that LCNPs and CLCNPs were extensively internalized in macrophage cells with strong green fluorescence (Fig. 3a). However, there were a weaker green fluorescence signals for CPLCNPs and PLCNPs in RAW 264.7 cells. The FACS analysis showed that the internalization of CPLCNPs and PLCNPs were reduced about 10 times when compared with the LCNPs group (Fig. 3b and c), illustrating that the PLT membrane camouflaged CPLCNPs and PLCNPs could obviously reduce the internalization in macrophage.

The effect of cancer cell-PLT hybrid membrane camouflage on cellular uptake was further assessed in Huh-7 cells by visualized LCSM and quantified FACS analysis. The LCSM images showed that CPLCNPs and CLCNPs displayed a higher internalization into Huh-7 cells than PLCNPs or LCNPs, which was denoted by the stronger green fluorescence signals (Fig. 3d). Moreover, the FACS analysis showed the cellular uptake of CPLCNPs in Huh-7 cells had a 1.8-fold higher signal than that of LCNPs (Fig. 3e and f), which effectively verified the enhanced effect of cancer cell membrane on CPLCNPs uptake by Huh-7 cells.

### In vitro* cytotoxicity and apoptosis studies*

**Fig. 4:**
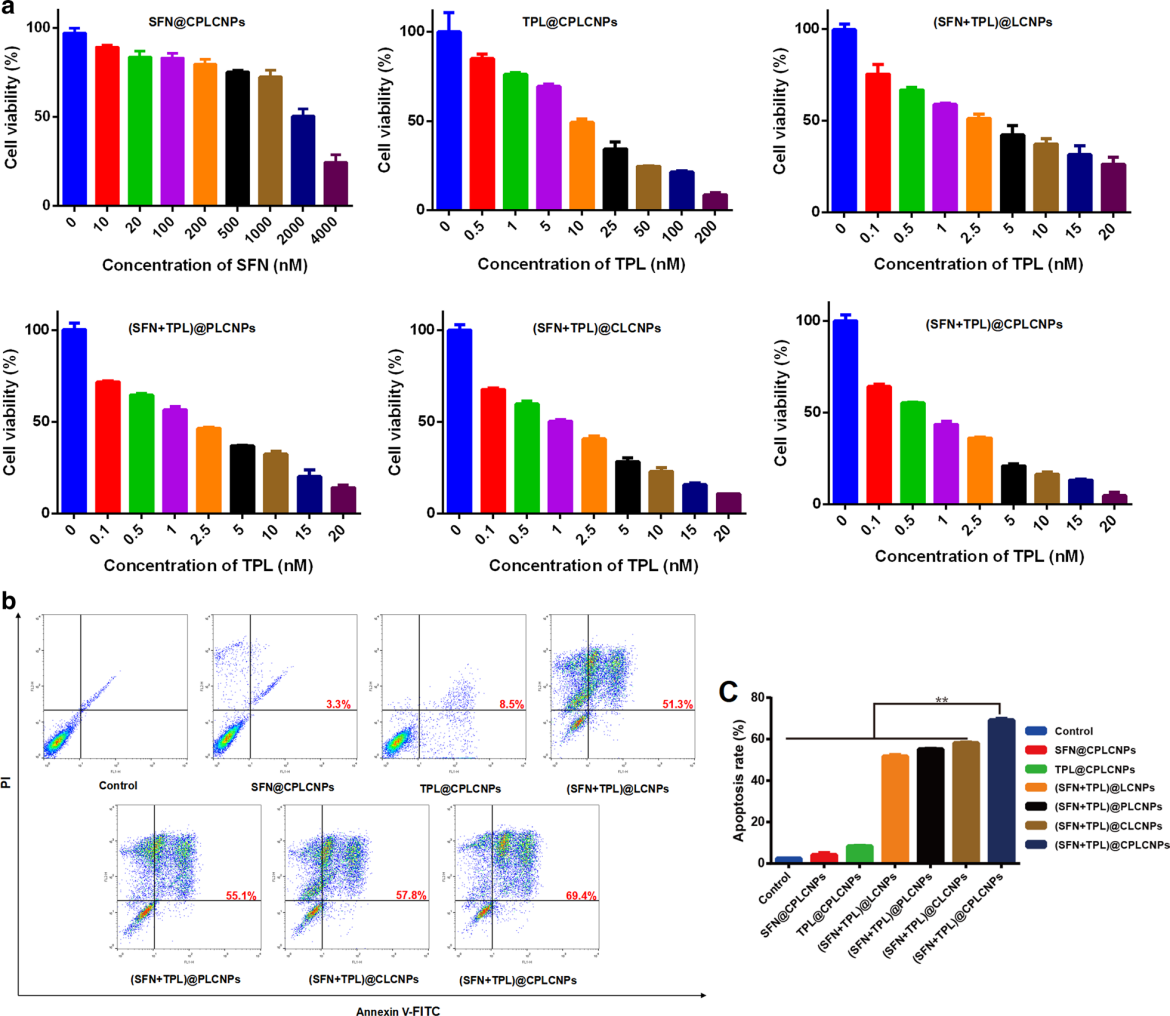
In vitro anti-tumor activity of different formulations: **a** cell viability of Huh-7 cells after treated with different formulations; **b** induction of apoptosis in Huh-7 cells treated with various formulations tested by flow cytometer, **c** the statistical results of Huh-7 cell apoptosis rate (n = 3; **p < 0.01)

The in vitro cytotoxicity of drug-loaded nanoparticles against Huh-7 cells were detected. After incubation for 24 h, all formulations demonstrated dose-dependent inhibitory activities against Huh-7 cells (Fig. 4a). Among all drug formulations, (SFN + TPL)@CPLCNPs exhibited the highest cytotoxicity at all tested concentrations. Furthermore, the concentrations of SFN and TPL at a 50% inhibition rate against Huh-7 cells for different formulations and the corresponding IC_50_ values were summarized in Table 1. Notably, (SFN + TPL)@LCNPs.

**Table 1:** The concentrations of SFN and TPL at a 50% inhibition rate against Huh-7 cells for different formulations and the corresponding CI (n = 3)

Formulations	Concentration of SFN (nM)	Concentration of TPL (nM)	CI_50_
SFN@CPLCNPs	2350.93		
TPL@CPLCNPs		8.91	
(SFN + TPL)@LCNPs	22.49	2.25	0.26
(SFN + TPL)@PLCNPs	12.03	1.20	0.14
(SFN + TPL)@CLCNPs	7.61	0.76	0.09
(SFN + TPL)@CPLCNPs	5.44	0.54	0.06

(SFN + TPL)@PLCNPs, (SFN + TPL)@CLCNPs, and (SFN + TPL)@CPLCNPs could lead to stronger cell inhibition effects compared with the free drug combination (Additional file 1: Table S1) and single drug-loaded groups, which might be due to the coordinated cellular uptake profiles of the two loaded drugs by tumor cells. The IC_50_ of SFN@CPLCNPs and TPL@CPLCNPs groups were 432.2- and 16.5-fold greater than that of (SFN + TPL)@CPLCNPs group, respectively. The enhanced cytotoxicity indicated that tumor-targeted nanoparticles could effectively and simultaneously transport different drug molecules into Huh-7 cells. Furthermore, as calculated with CompuSyn software, the CI_50_ values of SFN and TPL co-loaded nanoparticles against Huh-7 cells were all smaller than 1 at a SFN/TPL ratio of 10:1 with significant synergistic antitumor efficacy (Table 1). It was worth noting that the (SFN + TPL)@CPLCNPs group had the lowest CI_50_ value among the SFN and TPL co-loaded nanoparticles, indicating that cancer cell-PLT hybrid membrane played an important role in the enhancement of synergistic effects.

To further investigate the ability of the synergistic effects of SFN and TPL to induce apoptosis in Huh-7 cells, the Annexin V-FITC/PI method was adopted. Annexin V-FITC/PI double staining showed the highest percentage of early and late apoptotic cells in (SFN + TPL)@CPLCNPs group with the apoptosis rate at 69.4%, which was significantly higher than other tested groups (Fig. 4b and c). The results demonstrated that the combination of SFN and TPL could enhance the cell apoptosis effect, and this effect could be further enhanced by cancer cell-PLT hybrid membrane camouflaged nanoparticles.

### In vivo* biodistribution of homologous-targeting (SFN* + *TPL)@CPLCNPs*

**Fig. 5:**
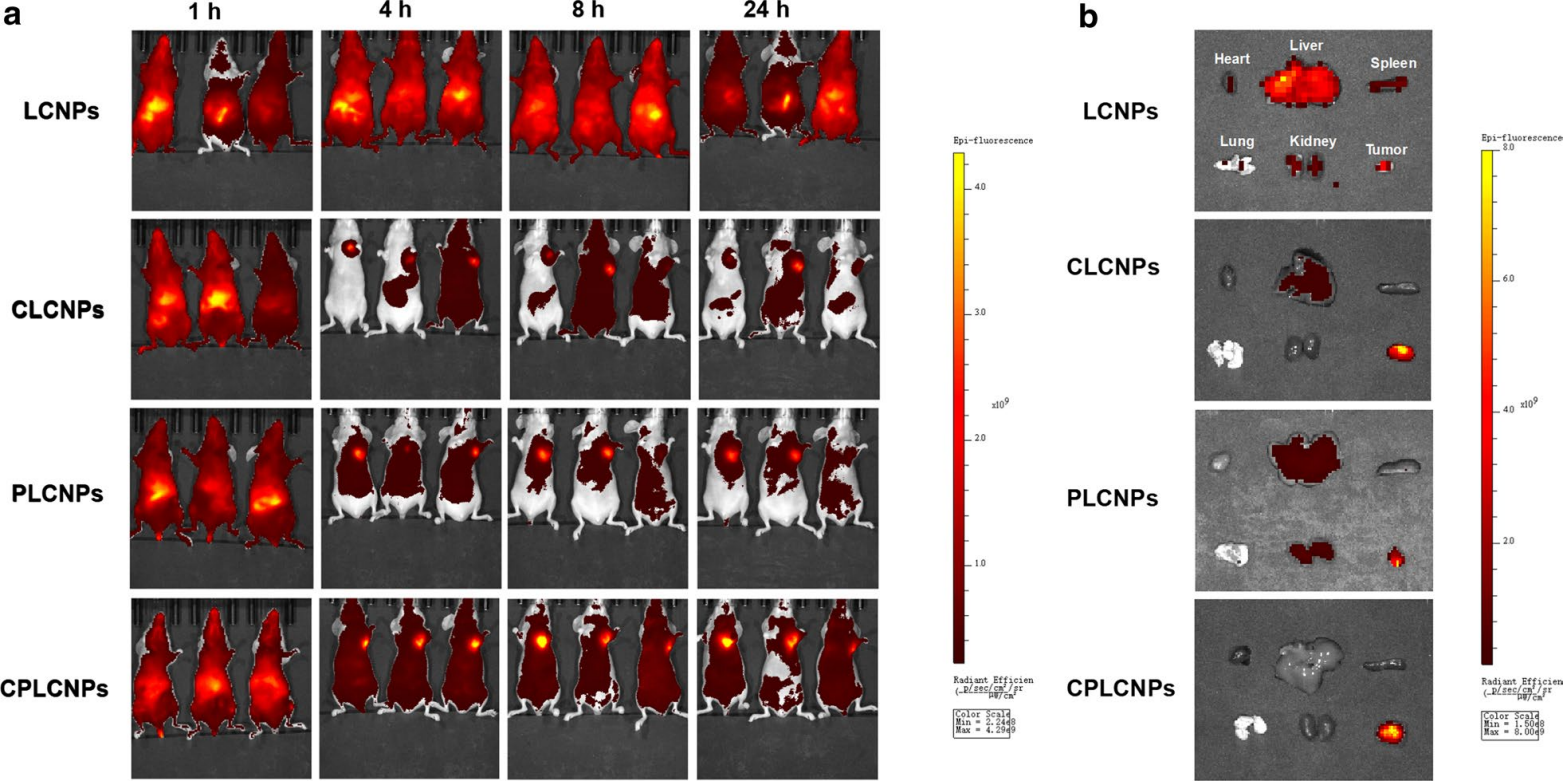
**a** Fluorescence images of tumor-bearing Balb/c-nu mice at different time points after the intravenous injection of various Cyanine 5.5 NHS ester-labled formulations, **b** fluorescence images of the removed tissues

Cyanine 5.5 NHS ester labeled nanoparticles were prepared. After injection through the tail vein, the distribution and accumulation of nanoparticles at the tumor sites of the tumor-bearing mice were observed on a real-time in vivo fluorescence animal imager. As shown in Fig. 5a, strongest fluorescence signals of Cyanine 5.5 NHS ester labeled CPLCNPs could be observed in tumor tissues at 8 h after injection, while tumor tissues treated with Cyanine 5.5 NHS ester labeled LCNPs, PLCNPs and CLCNPs exhibited weaker fluorescence signals, which indicated that CPLCNPs camouflaged with cancer cell-PLT hybrid membrane could exert its homologous tumor targeting and long circulation effects, leading to more nanoparticles concentrating in tumor tissues [22]. As for removed tissues (Fig. 5b), the fluorescence in the CPLCNPs group was also observably the highest in the tested groups at the tumor site. Thus, (SFN + TPL)@CPLCNPs could have superior anti-tumor effect than (SFN + TPL)@LCNPs, (SFN + TPL)@CLCNPs or (SFN + TPL)@PLCNPs.

### In vivo* anti-tumor activity of (SFN* + *TPL)@CPLCNPs*

**Fig. 6:**
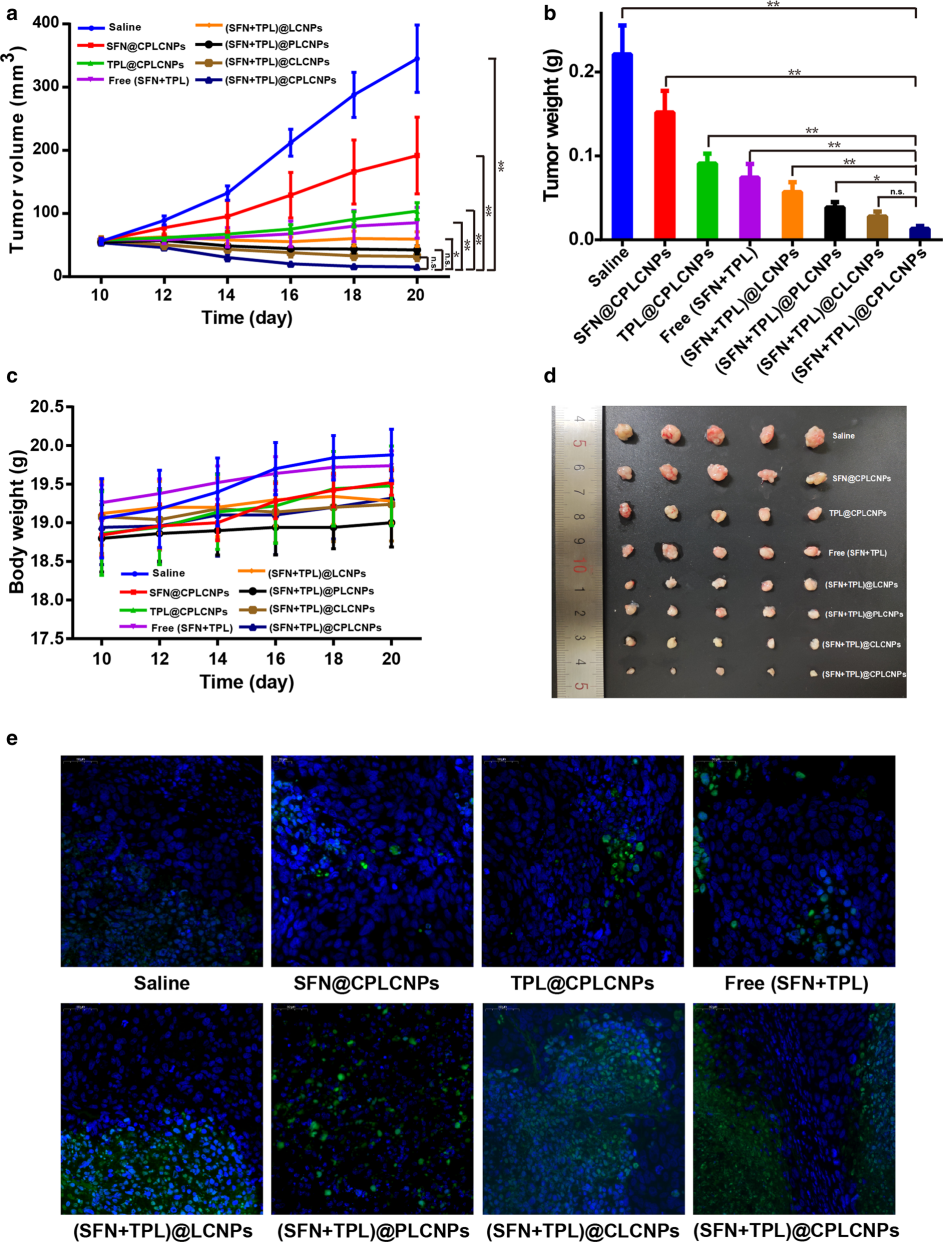
In vivo anti-tumor activity: **a** tumor volume, **b** tumor weights, **c** body weights changes, **d** photographs of tumors and **e** TUNEL staining of tumor-bearing Balb/c-nu mice after treated with various formulations (n = 5; **p < 0.01; *p < 0.05)

The in vivo anti-tumor activity of (SFN + TPL)@CPLCNPs was evaluated in Huh-7 tumor-bearing Balb/c-nu mice. The results indicated that continuous tumor growth was observed for the mice treated with saline and free (SFN + TPL), likely ascribing to the insufficient SFN and TPL retention in the tumor sites (Fig. 6a). For mice treated with SFN@CPLCNPs and TPL@CPLCNPs, tumor growth was also witnessed, but for mice receiving (SFN + TPL)@LCNPs, (SFN + TPL)@PLCNPs, (SFN + TPL)@CLCNPs and (SFN + TPL)@CPLCNPs, the tumors showed significantly inhibited growth and the (SFN + TPL)@CPLCNPs group showed slowest growth, smallest volume and lightest tumor weight at the end of the treatment (Fig. 6a, b, and d), manifesting that the combination of SFN and TPL had synergistic anti-tumor effects and the effects were further enhanced by cancer cell-PLT hybrid membrane camouflaged nanoparticles. Moreover, the body weights of the tumor-bearing mice did not change significantly during and after the treatment, demonstrating that the formulations did not produce significant toxicity when exerting a therapeutic effect (Fig. 6c). These data suggested that (SFN + TPL)@CPLCNPs had potent anti-tumor activity without obvious toxicity. In addition, in situ TUNEL assay (Fig. 6e) showed no green signals in the tumors of mice treated with saline, indicating the cells all to be viable. Some green signals are noticeable with all the drug treatments, indicative of apoptosis. The highest levels of green signals were seen in the tumors derived from mice treated with (SFN + TPL)@CPLCNPs, demonstrating that tumors in (SFN + TPL)@CPLCNPs group had the largest proportion of apoptotic tumor cells. Relevant study showed that SFN might inhibit the RAF/MEK/ERK signaling pathway, whereas TPL might inhibit the Akt/mTOR pathway and basal NF-kB activity/activation. Combined together, the two drugs acted on different pathways to enhance anti-tumor effects [3].

## Conclusions

In summary, we have successfully constructed a biomimetic nanosystem based on cancer cell-PLT hybrid membrane camouflage to co-deliver SFN and TPL using PLT membrane with long circulation functions and tumor cell membrane with homologous targeting. It was demonstrated that the cancer cell membrane and PLT membrane were camouflaged onto the LCNPs. (SFN + TPL)@CPLCNPs could load SFN and TPL at the molar ratio of SFN to TPL close to 10:1. The release of SFN and TPL from (SFN + TPL)@CPLCNPs exhibited slower release behaviour than LCNPs at 0.25, 0.5, 1, 2 and 8 h and maintain the simultaneous releasing profile, (SFN + TPL)@CPLCNPs achieved long circulation function and tumor targeting at the same time, promoting tumor cell apoptosis, inhibiting tumor growth, and achieving a better "synergy and attenuation effect". Taken together, this new hybrid membrane–camouflaged biomimetic nanosystem has the potential to provide a practical and innovative treatment for the treatment of HCC.

## Supplementary Information

**Additional file 1: Fig. S1**. Stability of (SFN+TPL)@LCNPs and (SFN+TPL)@CPLCNPs in plasma (n=3). As shown in Fig. S1, the particle size of (SFN+TPL)@LCNPs and (SFN+TPL)@CPLCNPs did not change significantly after 72 h in plasma, and still remained stable, indicating that the preparation had good stability and no precipitation or aggregation occurred. **Table S1**. The concentrations of SFN and TPL at a 50% inhibition rate against Huh-7 cells for different combinations and the corresponding CI (n=3)

## Data Availability

All data generated or analyzed during this study have been included in the article.
