# Supplementary material for: Sorafenib and triptolide loaded cancer cell-platelet hybrid membrane-camouflaged liquid crystalline lipid nanoparticles for the treatment of hepatocellular carcinoma
Source: J Nanobiotechnology. 2021 Nov 8;19:360. doi: 10.1186/s12951-021-01095-w (PMC8576878; doi:10.1186/s12951-021-01095-w)
Supplement: Supplementary file 1 — Additional file 1: Fig. S1. Stability of (SFN+TPL)@LCNPs and (SFN+TPL)@CPLCNPs in plasma (n=3). As shown in Fig. S1, the particle size of (SFN+TPL)@LCNPs and (SFN+TPL)@CPLCNPs did not change significantly after 72 h in plasma, and still remained stable, indicating that the preparation had good stability and no precipitation or aggregation occurred. Table S1. The concentrations of SFN and TPL at a 50% inhibition rate against Huh-7 cells for different combinations and the corresponding CI (n=3) [file 12951_2021_1095_MOESM1_ESM.docx]

**Supplementary material**

**

**

**Fig. S1** Stability of (SFN+TPL)@LCNPs and (SFN+TPL)@CPLCNPs in plasma (n=3)

As shown in Fig. S1, the particle size of (SFN+TPL)@LCNPs and (SFN+TPL)@CPLCNPs did not change significantly after 72 h in plasma, and still remained stable, indicating that the preparation had good stability and no precipitation or aggregation occurred in.

**Table S1.** The concentrations of SFN and TPL at a 50% inhibition rate against Huh-7 cells for different combinations and the corresponding CI (n=3)

| Formulations | Concentration of SFN (nM) | Concentration of TPL (nM) | CI_50_ |
| --- | --- | --- | --- |
|  |  |  |  |
| Free SFN | 8797.41 |  |  |
| Free TPL |  | 13.06 |  |
| SFN:TPL=1:1 | 69.84 | 69.84 | 5.36 |
| SFN:TPL=5:1 | 124.15 | 25.23 | 1.95 |
| SFN:TPL=10:1 | 71.67 | 7.17 | 0.56 |
| SFN:TPL=15:1 | 180.88 | 12.06 | 0.94 |
| SFN:TPL=25:1 | 406.58 | 16.26 | 1.29 |
| SFN:TPL=40:1 | 5511.98 | 137.80 | 11.18 |
| SFN:TPL=50:1 | 715.46 | 14.31 | 1.18 |

When the molar ratio of SFN and TPL was at 10:1, the CI_50_ was lowest. As a result, SFN and TPL with molar ratio at 10:1 was selected as best drug combination for the treatment of HCC.
